# Contacting individual graphene nanoribbons using carbon nanotube electrodes

**DOI:** 10.1038/s41928-023-00991-3

**Published:** 2023-08-14

**Authors:** Jian Zhang, Liu Qian, Gabriela Borin Barin, Abdalghani H. S. Daaoub, Peipei Chen, Klaus Müllen, Sara Sangtarash, Pascal Ruffieux, Roman Fasel, Hatef Sadeghi, Jin Zhang, Michel Calame, Mickael L. Perrin

**Affiliations:** 1 Transport at Nanoscale Interfaces Laboratory, Empa, Swiss Federal Laboratories for Materials Science and Technology, Dübendorf, Switzerland; 2 College of Chemistry and Molecular Engineering, Peking University, Beijing, China; 3 nanotech@surfaces Laboratory, Empa, Swiss Federal Laboratories for Materials Science and Technology, Dübendorf, Switzerland; 4 School of Engineering, University of Warwick, Coventry, UK; 5 Nanofabrication Laboratory, National Center for Nanoscience and Technology, Beijing, China; 6 Max Planck Institute for Polymer Research, Mainz, Germany; 7 Department of Chemistry, Biochemistry and Pharmaceutical Sciences, University of Bern, Bern, Switzerland; 8 Department of Physics, University of Basel, Basel, Switzerland; 9 Swiss Nanoscience Institute, University of Basel, Basel, Switzerland; 10 Department of Information Technology and Electrical Engineering, ETH Zurich, Zurich, Switzerland; 11 Quantum Center, ETH Zurich, Zurich, Switzerland

**Keywords:** Electronic devices, Electronic properties and materials, Electrical and electronic engineering, Graphene, Carbon nanotubes and fullerenes

## Abstract

Graphene nanoribbons synthesized using bottom-up approaches can be structured with atomic precision, allowing their physical properties to be precisely controlled. For applications in quantum technology, the manipulation of single charges, spins or photons is required. However, achieving this at the level of single graphene nanoribbons is experimentally challenging due to the difficulty of contacting individual nanoribbons, particularly on-surface synthesized ones. Here we report the contacting and electrical characterization of on-surface synthesized graphene nanoribbons in a multigate device architecture using single-walled carbon nanotubes as the electrodes. The approach relies on the self-aligned nature of both nanotubes, which have diameters as small as 1 nm, and the nanoribbon growth on their respective growth substrates. The resulting nanoribbon–nanotube devices exhibit quantum transport phenomena—including Coulomb blockade, excited states of vibrational origin and Franck–Condon blockade—that indicate the contacting of individual graphene nanoribbons.

## Abstract (web summary)

Individual graphene nanoribbons synthesized by an on-surface approach can be contacted with carbon nanotubes—with diameters as small as 1 nm—and used to make multigate devices that exhibit quantum transport effects such as Coulomb blockade and single-electron tunnelling.

## Main

Bottom-up synthesized graphene nanoribbons (GNRs) are a tunable class of quantum material with a wide range of electronic, magnetic and optical properties, including variable bandgaps, single-photon emission and spin-polarized/topologically protected states^1–5^. Such materials offer greater chemical flexibility than those fabricated using top-down approaches^6^, where control over width and edge morphology is limited and can lead to additional localized states induced by disorder at the edges. Using the materials to make quantum devices requires control over their chemical structure, and their integration into device architectures^7,8^. The integration and contacting of an individual GNR with atomic precision could, for example, be used to create semiconducting quantum dots (QDs) that trap individual charges and their associated spins. These could be used to create charge or spin qubits, as well as single-photon emitters.

**Fig. 1:**
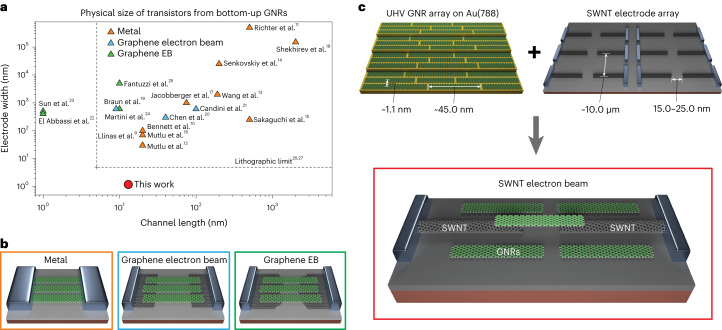
Size scaling in bottom-up GNR-based transistors with various geometries. **a**, Comparison of the physical size of transistors from GNRs with different contact strategies: metal electrodes^9–18^ (orange), EBL-defined graphene electrodes^19–21^ (blue), EB-formed graphene electrodes^22–25^ (green) and EBL-defined SWNT electrode (red; this work). The squares represent surface-polymerized GNRs in an ultrahigh vacuum; the triangles represent solution-polymerized GNRs; the circles represent CVD-synthesized GNRs. **b**, Schematic of the transistors of typical bottom-up GNR transistors with metal electrodes (left), EBL-defined graphene electrodes (middle) and EB-formed graphene electrodes (right). **c**, Ultimately scaled SWNT electrodes for contacting bottom-up GNRs. Schematic of the ultrahigh vacuum (UHV)-synthesized GNR array parallel to the Au(788) terraces (top left). Schematic of the parallel SWNT electrode array on a SiO_2_ substrate (top right). Schematic of a single-GNR-based transistor with SWNTs as ultimately scaled electrodes (bottom). For clarity, only the GNRs closest to the nanogap are shown.

Contacting individual GNRs—particularly on-surface synthesized ones—is, however, a challenging task^5,7,8^. Bottom-up synthesized GNRs have previously been contacted using different approaches (Fig. 1a,b), with the electrode material either a noble metal (gold, platinum or palladium)^9–18^ or graphene^19–25^. Electrodes can be fabricated before or after the GNR transfer, referred to as ‘GNR-last’ and ‘GNR-first’ approaches, respectively. The GNR-first approach is the preferred method for ultrashort channel lengths when metallic electrodes are used^9–18^. The metallic electrodes are created using electron-beam lithography (EBL) techniques that can cause contamination and damage to the GNRs during the fabrication process (Fig. 1b, left). Graphene is an appealing alternative because it is naturally atomically flat, making it optimally suited for the GNR-last approach. Graphene electrodes are either defined using EBL-defined nanogaps^19–21^ (Fig. 1b, middle) or formed using the electrical breakdown (EB) method that results in ultranarrow nanogaps in the range of 1–5 nm (refs. ^22–25^) (Fig. 1b, right).

However, for both metallic and graphene electrodes, it is still challenging to contact an individual GNR because of their intrinsically small width and lateral separation (Fig. 1a), typically of the order of 1–2 nm, which is below the capabilities of state-of-the-art EBL^26,27^ (Fig. 1a; grey dashed lines). The inter-ribbon separation distance between the GNRs could instead be increased, but this is usually achieved by reducing the amount of precursor molecules on the growth substrate and leads to shorter GNRs^28^.

Individual GNRs have previously been contacted using graphene-based breakdown gaps. However, this method yields ill-defined electrode geometries and only works for very short GNRs that are comparable with electrode separation (around 5 nm). Longer GNRs allow for the creation of superlattices in which localized states or spins are periodically placed along the GNRs, making it possible to engineer spin chains^29^ or topologically protected states^4^. In such GNRs, it may be desirable to have the functional part located in between the electrodes, rather than on top of the electrodes. However, as the GNR lengths increase, the probability of the electrodes bridging multiple GNRs also increases. Moreover, the lack of precise control over the nanogap location prevents creating devices with multiple gates, which is required for the control of multi-QD systems.

Currently, long GNRs have only been contacted using graphene or metal electrodes where bridging of multiple GNRs, both in parallel and series, is likely. This leads to the formation of irregular and non-closing Coulomb diamonds^30^, making the exploitation of the electronic structure of a single GNR for device applications challenging. Therefore, alternative contacting methods for long GNRs, such as one-dimensional electrodes^31^, are required.

In this Article, we report the contacting of individual on-surface synthesized long GNRs in a multigate transistor geometry using single-walled carbon nanotube (SWNT) electrodes (Fig. 1c). Our approach relies on the self-aligned nature of both SWNTs, which have diameters as small as 1 nm, and GNR growth on their respective growth substrate. The assembly of SWNT–GNR–SWNT devices is verified from the spectroscopy data of the molecular levels performed at cryogenic temperature, which shows several features that are characteristic of transport through an individual GNR, such as Coulomb blockade, presence of vibrational modes in the single-electron tunnelling (SET) regime and Franck–Condon blockade. The multiple gates also allow the conductivity of the GNRs and SWNT electrodes to be individually tuned, as well as for the origin of the different states observed in the spectroscopic measurements to be identified. The ability to contact long GNRs precisely in a multigate architecture could enable the control of double- or multiple-QD systems in the future.

## Device design

**Fig. 2:**
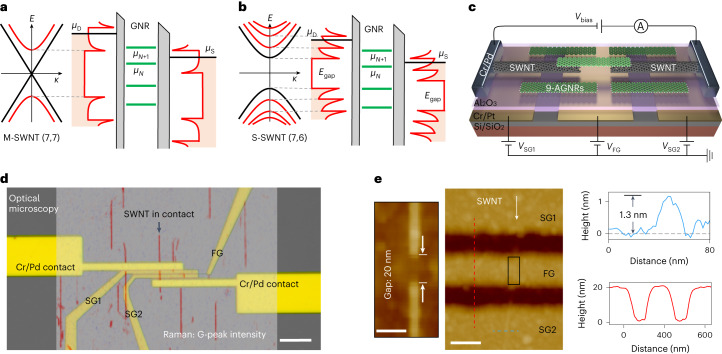
Multigate 9-AGNR transistors with SWNT electrodes. **a**, Electronic dispersion relation of a representative M-SWNT (7,7) with sub-bands and zero bandgaps (left). Illustration of the discrete energy levels of the GNR and sharp DOS in the two SWNT electrodes (right). The sharp DOS peaks exhibit Van Hove singularities, which are associated with the sub-bands of the SWNT. Note that we indicate the barriers between the left SWNT electrode and GNR, and between the right SWNT electrode and GNR. The asymmetrical barriers are illustrated in practice. **b**, Similar illustration as **a** for a representative S-SWNT (7,6) with the same diameter as the M-SWNT in **a**. It has relatively more dense Van Hove singularities in the DOS and a finite bandgap. The electronic dispersion and DOS for SWNTs (7,7) and (7,6) are adapted from another work^33^. Note that the chiralities of the SWNTs used for this work were not determined. **c**, Schematic of the device, including the measurement circuit. **d**, Optical image of a device with an overlay of the G-peak Raman intensity map coloured in red (532 nm laser, 1 mW power and 1 s integration time). Scale bar, 10 μm. **e**, Topographic AFM image showing the SWNT electrodes and gate layout of the device. High-resolution AFM characterization of a representative SWNT nanogap (~20 nm) defined by EBL (left). Scale bar, 20 nm. Topography profile across the SWNT (blue solid line) and gate electrodes (red solid line) with a 30 nm atomic-layer-deposited Al_2_O_3_ layer on top (middle). Scale bar, 100 nm. Profiles (blue and red solid lines) are taken along the blue and red dotted lines in the corresponding AFM image, respectively (right).

The studied devices (Fig. 2) consist of a pair of SWNT electrodes separated by 15–25 nm. Below the nanogap, a 100-nm-wide Cr/Pt finger gate (FG) is fine patterned alongside the two side gates (SG1 and SG2). Multiple gates are required for controlling the density of states (DOS) of the SWNT leads. Due to quantum confinement of the charge carriers as a result of the one-dimensional nature of the SWNTs, sharp peak-like Van Hove singularities appear at the onset of each sub-band^32,33^. In addition, SWNTs come in two types: metallic SWNTs (M-SWNTs) and semiconducting SWNTs (S-SWNTs). Although M-SWNTs exhibit a flat and non-zero DOS around the Fermi energy, their semiconducting counterparts have a sizable bandgap. Figure 2a,b illustrates the band diagrams of the SWNT–GNR–SWNT junctions with the discrete energy levels of the GNR and the Van Hove singularities in the DOS of the M-SWNT and S-SWNT leads, respectively. The multiple gates are separated from the junction by a 30-nm-thick Al_2_O_3_ layer. A film of GNRs is then transferred on top of the device substrate. Figure 2c shows a schematic of the device. A detailed description of the materials and fabrication process is provided in Methods and Supplementary Section 1.

Figure 2d shows an optical image of a representative device, with three gates and source/drain contacts. The Raman intensity map of the G peak is presented as a red overlay in this figure, highlighting the presence of the uniaxially aligned SWNT array, with a single SWNT bridging the metallic source/drain contacts. Figure 2e shows an atomic force microscopy (AFM) image of the device focusing on the gate structure and SWNT electrodes. A high-resolution AFM image of the SWNT nanogap is presented in the inset of this figure, showing a gap size of ~20.0 nm and SWNT diameter of 1.3 nm. Supplementary Section 1.1 shows more characterizations of the SWNT diameters. As the SWNT diameter is of a similar size as the GNR width, we anticipate that one—or at the most, two—GNRs can make contact to a pair of SWNT electrodes.

In total, eight chips were characterized, five with the multigate architecture and three with a single back gate, for a total of ~2,500 devices (this number only includes devices on which GNRs were transferred, not those present on the chips but located outside the area covered by the GNR films). Among them, ~600 SWNT transistors were functional at room temperature, as assessed by electrical characterization, before the nanogap formation. After the nanogap formation, 360 devices showed clearly separated SWNT electrodes, with currents lower than 10 pA at 1 V. After the GNR transfer, 41 of those devices showed a gate-modulated current. GNR films are known to conduct at room temperature, and therefore, these devices cannot be solely attributed to nanogaps containing only individual GNRs. However, as film transport is temperature activated, it is easily suppressed by cooling down the sample to cryogenic temperatures. At temperatures below 9 K, 12 devices showed QD behaviour. This corresponds to a yield of 3.3% when considering only the number of nanogaps that were well formed before the GNR transfer. Methods provides details about the electrical characterization. Supplementary Section 1.3 provides the typical current–voltage characteristics and gate sweep at room temperature, showing that the GNRs behave as p-type semiconductors, in agreement with previous results^19^.

In the following sections, we discuss QD devices based on M-SWNT leads (devices D3 and D6) and S-SWNT leads (D7), all of which were obtained using the multigate architecture and characterized at a base temperature of 255 mK using a ^3^He system. Additional devices, either based on the global back-gate (Supplementary Section 2; D1) or multigate (Supplementary Section 3; D4, D5 and D8) architecture, are presented in the Supplementary Information.

## Multigate devices with M-SWNT leads

**Fig. 3:**
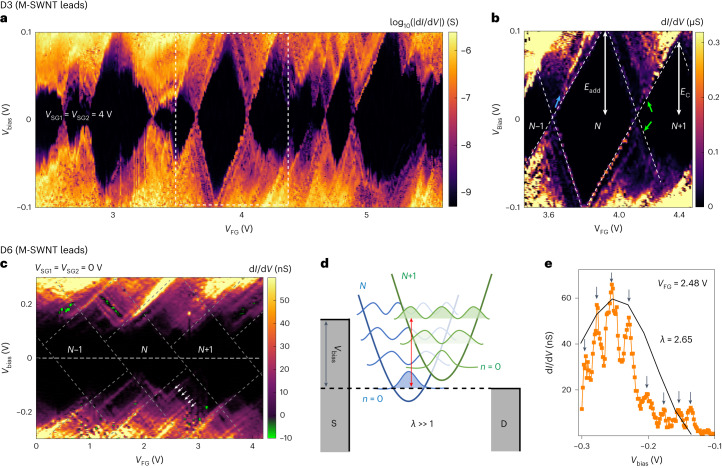
Electron transport in 9-AGNR transistors (D3 and D6) with M-SWNT leads. **a**,**b**, Single-electron charging behaviour in D3. Colour-scaled differential conductance versus *V*_FG_ and *V*_Bias_ at fixed *V*_SG1_ = *V*_SG2_ = 4 V, showing single-electron charging behaviour (**a**). Close-up of the box in **a** (white dotted line), highlighting the excited states (green arrows) and lead states (blue arrow) (**b**). **c**–**e**, Franck–Condon blockade in D6. Colour-scaled differential conductance versus *V*_FG_ and *V*_Bias_ for *V*_SG1_ = *V*_SG2_ = 0 V (**c**). Low-bias conductance is suppressed and the Coulomb blockade cannot be lifted by *V*_FG_. Periodic excitations (white arrows) appear within the conductive regime at positive and negative biases. NDC appears in some regions (green colour). **d**, Schematic of the Franck–Condon model for strong electron–phonon coupling *λ*, with *N* (blue curve) and *N* + 1 (green curve) electrons in the QD. The tunnelling electron shifts the equilibrium coordinate of the phonon harmonic oscillator by an amount proportional to *λ*, thereby exponentially suppressing the transition between the vibrational ground states of the *N* and *N* + 1 charge states. **e**, Differential conductance measured for *V*_FG_ = 2.48 V and *V*_SG1_ = *V*_SG2_ = 0 V. The representative fit of the maxima with the Franck–Condon progression (equation (1)) enables us to extract the coupling as *λ* = 2.65.

Figure 3a,b presents the transport data for D3 with a pair of M-SWNT leads. Figure 3a shows the differential conductance (d*I*/d*V*) as a function of *V*_FG_ and *V*_Bias_ (the so-called stability diagram) for fixed side-gate voltages of *V*_SG1_ = *V*_SG__2_ = 4 V. For the given gate voltage range, several Coulomb diamonds are observed with strong variations in the addition energies, ranging from 33 to 110 meV. Although for top-down GNRs, the strong variation in addition energies may be a sign of disorder caused by localized states in the edges, bottom-up GNRs naturally possess this variation as a result of strong quantum confinement^6^. A close-up of the boxed region in Fig. 3a (Fig. 3b) shows a well-resolved SET regime with multiple resonances that run parallel to the edge of the diamond (Fig. 3b, green arrows). For the SET regime around a gate voltage of 4 V, the excited states at positive and negative biases are located at 25 mV and –23 mV (Fig. 3b, green arrows), respectively. We attribute these resonances to the presence of vibrational modes in the nine-atom-wide armchair graphene nanoribbons (9-AGNRs), which are discussed further later. To confirm the conductive nature of the electrodes due to the absence of a bandgap, we measured the stability diagram in a different transport regime by applying side-gate voltages of *V*_SG1_ = *V*_SG2_ = 0 V, yielding qualitatively similar results (Supplementary Section 3.1).

In addition to the excited states, we observe additional resonances that we attribute to the modulation of the DOS in the SWNT leads^34^ (Fig. 3b, blue arrow). These states can be distinguished by their slope Δ*V*_Bias_/Δ*V*_FG_ in the stability diagram, which is different from the slopes of the edge of the SET regime. The origin of such a slope difference is the different gate couplings of the FG to GNRs and SWNT leads. Additionally, we observe several Coulomb diamonds (Fig. 3a) that do not have a crossing point at each of their sides, for example, at *V*_FG_ of ~3.0, ~4.4 and ~5.4 V. This may be due to the mixing of the lead states with QD states^35^, but may also come from disorder—either intrinsic to the GNR or induced by the environment^6^. Although for top-down GNRs, a substantial portion of the disorder originates from the uncontrolled edges that possess localized edge states, bottom-up 9-AGNRs are atomically precise and do not have such localized edge states. However, disorder from the environment (such as residues, possible defects in the SWNT electrodes, charge traps in the oxide and so on) is very challenging to avoid and may explain the few non-closing and/or deformed diamonds. Supplementary Section 3.2 discusses two additional devices (D4 and D5) with M-SWNT leads are shown, with a qualitatively similar behaviour.

Figure 3c–e shows the transport data for D6 with a pair of M-SWNT leads. Although D6 has a similar fabrication process as D3, D4 and D5, richer physics is observed. Figure 3c shows a stability diagram for fixed *V*_SG1_ = *V*_SG2_ = 0 V. In the given *V*_FG_ ranges, Coulomb diamonds are observed, possessing several notable features. First, quasi-periodic lines (Fig. 3c, white arrows) running parallel to the edges of the Coulomb diamonds are observed when *V*_Bias_ > 60 meV and *V*_Bias_ < –60 meV. The energy spacing between these excited states is Δ*E* = 29 meV on average. Second, conductance is highly suppressed in the low-bias regime approximately between +60 and –60 meV present in all the probed diamonds. Third, in some regions, negative differential conductance (NDC) appears in between resonances (Fig. 3c, green). We attribute these three features to phonon-assisted tunnelling transport, enabled by a strong electron–phonon coupling in our GNR junction, as discussed in more detail below.

Similar quasi-periodic resonances have been previously attributed to the excitation of vibrational modes, as observed in single-molecule transistors^36–39^ and suspended SWNTs^40,41^. The zero-bias conductance suppression may originate from the Franck–Condon blockade effect^37,38,41,42^, which occurs in cases of strong electron–phonon coupling (*λ* ≫ 1) (Fig. 3d). Here sequential electron tunnelling is strongly suppressed due to the exponentially small overlap of the harmonic oscillator wavefunctions of the different charge states, and charge transport can only occur when the bias is large enough to overcome the phononic energy difference by exciting phonons. To extract the electron–phonon coupling *λ*, we study the d*I*/d*V* versus *V*_Bias_ at a fixed *V*_FG_ = 2.48 V (Fig. 3e). By fitting the maxima of d*I*/d*V* with the Franck–Condon model (Methods), *λ* is determined to be 2.65. The average *λ* value obtained from another four d*I*/d*V* traces at different transport regimes is 2.66 ± 0.09 (Supplementary Section 3.3). Overall, the *λ* value is symmetric with respect to the bias polarity and independent of the charge state, which is consistent with a phononic origin. Another interesting feature of the data is the appearance of the NDC (Fig. 3c). Such NDC regions have previously been associated with electron–phonon interactions, according to theoretical^43–45^ and experimental^40,41^ studies. Importantly, the three abovementioned transport features require a strong electron–phonon coupling, as well as the presence of a single QD in the junction area.

To determine the position of the QD along the SWNT–GNR–SWNT channel, we measured the current as a function of multiple gates and extracted the relative gate couplings as *α*_FG_:*α*_SG1_:*α*_SG2_ = 1.00:0.81:0.29. Supplementary Section 3.4 provides more details of the analysis of gate coupling. Based on this and the position of the gates with respect to the SWNT–GNR–SWNT channel, we conclude that the QD is formed in the GNR, rather than in the SWNT leads. Finally, we extract the electronic coupling of the GNR to the SWNT electrode and find values in the range of 4.9–7.7 meV. Supplementary Section 3.5 provides a description of how the coupling is obtained.

## Electron and phonon properties of 9-AGNRs

**Fig. 4:**
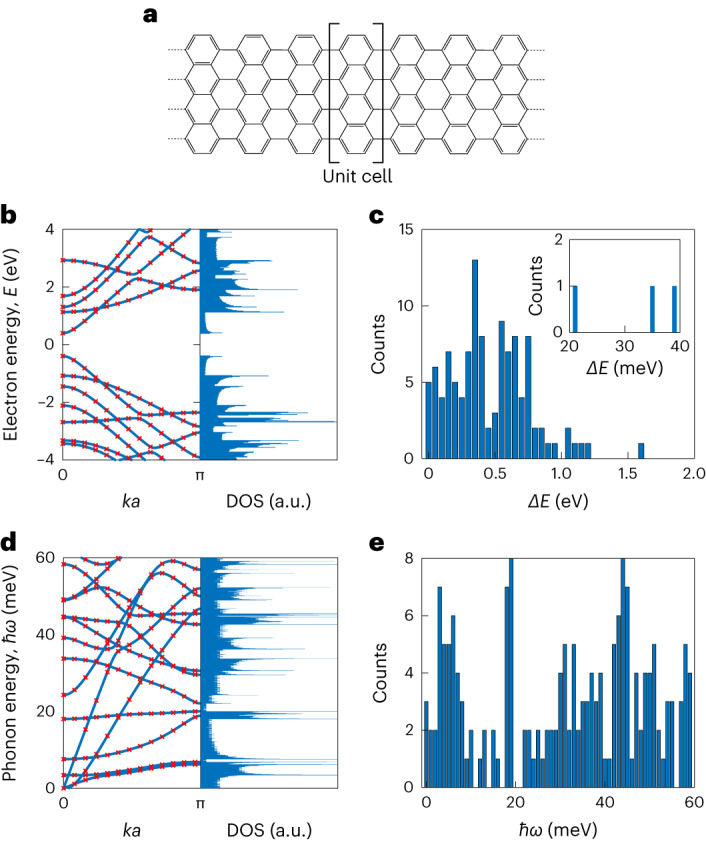
Electron and phonon properties of 9-AGNRs. **a**, Molecular structure of a 9-AGNR. **b**, Electron band structure of 9-AGNR and the corresponding DOS. The energy scale is within ±4 eV. **c**, Histogram of electron energy-level spacing of 9-AGNR determined by the energy differences of neighbouring bands at various *ka* values (red cross in **b**). The inset presents a zoomed-in view of the 20–40 meV region. **d**, Phonon band structure of 9-AGNR and the corresponding DOS. The energy scale is within 0–60 meV. **e**, Histogram of phonon energy of 9-AGNR determined by the energies of different bands at various *ka* values (red cross in **d**). The discretization of all the energy bands^57^ for *ka* = [0, π] is performed using Δ*ka* = 2π/*N* = 0.251, where *N* = 25.

To rationalize the charge transport measurements, particularly to identify the origin of the resonances observed in the SET regime of devices D3 and D6, we performed quantum chemistry calculations. We initially compute the electron and phonon band structures by performing periodic density functional theory (DFT) calculations of a 9-AGNR unit cell (Methods). To account for the quantum confinement effect in a finite-length 9-AGNR of 60 nm, we discretize the two band structures and obtain the corresponding energy levels^46^. Figure 4a shows a schematic of the 9-AGNR, alongside the calculated electronic band structure and DOS (Fig. 4b). The plot displays a semiconducting behaviour with a bandgap of ~796 meV, in agreement with previous electronic band structure calculations^47^. The red crosses on the band structure graph correspond to the discretized energy levels of the 9-AGNR. From the energy-level spectrum, we create a histogram of the energy differences of adjacent energy levels for the selected *ka* values (Fig. 4c).

**Fig. 5:**
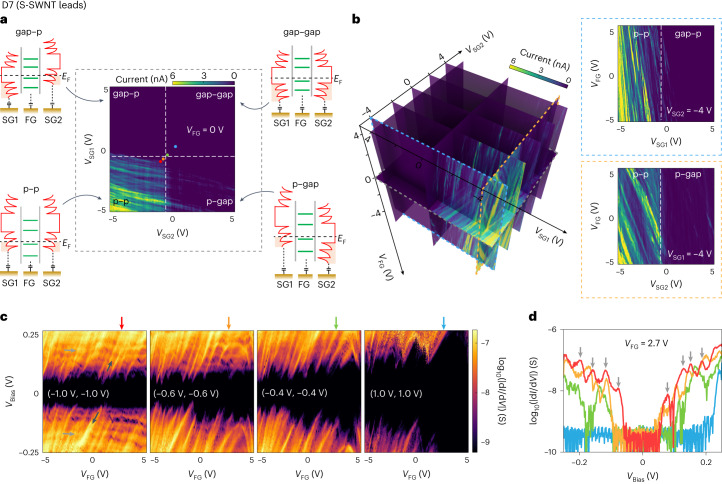
Electron transport in 9-AGNR transistors (D7) with S-SWNT leads. **a**, Colour-scaled current versus *V*_SG1_ and *V*_SG2_ at fixed *V*_Bias_ = 100 mV and *V*_FG_ = 0 V. The four sketches on the four corners illustrate the band diagrams of four different regimes for the SWNT electrodes (from left to right): p–p, p–gap, gap–p or gap–gap. The four coloured dots mark the positions of (*V*_SG1_, *V*_SG2_) settings for the measurements in **c**. **b**, Evolution of electrical transport (current) as a function of *V*_FG_, *V*_SG1_ and *V*_SG2_ (left). Nine colour-scaled current maps are shown in the three-dimensional plot: map of current versus *V*_SG1_ and *V*_SG2_ at *V*_FG_ = 0 V and *V*_Bias_ = 100 mV (also shown in **a**); maps of current versus *V*_FG_ and *V*_SG1_ at five different *V*_SG2_ values of –4, –2, 0, 2 and 4 V, and *V*_Bias_ = 50 mV; maps of current versus *V*_FG_ and *V*_SG2_ at three different *V*_SG1_ values of –4, 0 and 4 V, and *V*_Bias_ = 50 mV. Two example maps as edge highlighted by the blue dashed line and yellow dashed line in the left three-dimensional plot (right). Current versus *V*_FG_ and *V*_SG1_ values at *V*_SG2_ = –4 V (top right); current versus *V*_FG_ and *V*_SG2_ at *V*_SG1_ = –4 V (bottom right). **c**, Colour-scaled differential conductance versus *V*_FG_ and *V*_Bias_ for different (*V*_SG1_, *V*_SG2_) settings as per their position marked by the four coloured dots in **a**—from left to right: (–1.0 V, –1.0 V), (–0.6 V, 0.6 V), (–0.4 V, 0.4 V) and (1.0 V, 1.0 V). Several resonances with two different slopes are marked with grey or yellow arrows. **d**, Differential conductance versus *V*_Bias_ cut along *V*_FG_ = 2.7 V in the four maps shown in **c** (as marked by the four coloured arrows on top of the maps).

The plot shows that for all *ka* values combined, most of the energy spacings are on the order of hundreds of millielectronvolts up to electronvolts, with hardly any counts in the tens of millielectronvolt range, and no counts between 22 and 34 meV (Fig. 4c, inset). Similar energy spacings are also observed when the GNR is contacted with SWNT electrodes (Supplementary Section 4). Figure 4d shows the calculated phonon band structure and corresponding DOS for energies up to 60 meV, including the discretized values and a histogram of all the vibrational modes (Fig. 4e). The histogram possesses tens of modes in the 20–30 meV range, which is comparable with the experimentally observed values of 23–25 meV for device D3, and more than one order of magnitude smaller than the typical level spacings computed for electrons^40^ (Fig. 4c). From the absence of electronic energy spacings in the 22 to 34 meV range (Fig. 4c) and the dense population of vibrational modes in the same range, we attribute the excited states in device D3 to vibrational modes. This observation is also in line with the observed equidistant resonances and low-bias gap in device D6 being caused by Franck–Condon blockade. Indeed, the observed equidistant energy spacing Δ*E* (~29 meV) is consistent with the low-energy regime (20–40 meV) where many vibrational modes exist.

## Multigate devices with S-SWNT leads

We next studied devices with S-SWNTs, for which the electronic structure is expected to be substantially more tunable with side-gate voltage than for M-SWNTs. The transport measurements on device D7 with semiconducting electrodes are shown in Fig. 5. Figure 5a displays the current as a function of *V*_SG1_ and *V*_SG2_ for a fixed *V*_Bias_= 100 mV and *V*_FG_ = 0 V. This current map shows that the leads are conductive when a negative voltage (p side) is applied to either of the side gates, whereas transport is suppressed for a positive voltage. This large tunability is a direct consequence of the semiconducting nature of the SWNT electrodes. As a result, depending on the combination of SG voltages, the device can be tuned in four different transport regimes: p–p, p–gap, gap–p or gap–gap. Here, in each regime, either of the two electrodes is selectively switched off/on, as illustrated by the schematic of the four energy diagrams.

In addition to these four main regimes, some fine structure in the form of closely located, predominantly horizontal, resonances are observed in the p–p regimes (we note that some of them extend to the p–gap regime), as well as some weaker, vertical resonances. To investigate the origin of these resonances in more detail, Fig. 5b presents the evolution of the current and changing *V*_FG_, *V*_SG1_ and *V*_SG2_. This allows us to determine the coupling of each of the observed resonances to the different gates. For the *V*_FG_–*V*_SG1_ map at negative *V*_SG2_ of –4 V, a range of resonances is observed for negative voltages on SG1, with a much stronger coupling to SG1 than to FG. When gradually increasing *V*_SG2_, the resonances gradually fade out and at *V*_SG2_ = 4 V, most of them have disappeared. Similar resonances are observed in the *V*_FG_–*V*_SG__2_ maps, also with a stronger coupling to the side gate. From the much stronger coupling of the resonances to SG1 (SG2) than to FG, we conclude that these resonances originate from states in the S-SWNT electrodes.

To further investigate charge transport through the S-SWNT–GNR–S-SWNT device, we record maps of differential conductance versus *V*_Bias_ and *V*_FG_ for four combinations of (*V*_SG1_, *V*_SG2_) (Fig. 5c). The side-gate voltages at which the maps have been recorded are highlighted with coloured dots (Fig. 5a). The voltages were chosen such that the transport through the device gradually transitions from the p–p to the gap–gap regime of SWNT electrodes. Several characteristic features are observed in the d*I*/d*V* map of *V*_SG1_ = *V*_SG2_ = –1 V. For bias voltages below 50 mV, the current is suppressed. In the bias regime above 50 mV, current is flowing and two typical resonances are observed: first, resonances that are highly affected by *V*_FG_ (referred to as FG-dependent resonance, with a representative highlighted by the dark gray arrow in Fig. 5c); second, resonances that are mostly unaffected by FG (FG-independent resonance), resulting in predominantly horizontal lines in the d*I*/d*V* maps (Fig. 5d, light grey arrows). For *V*_SG1_ = *V*_SG2_ = –0.6 V, the low-bias gap slightly increases. Moreover, the FG-independent resonances in the negative-bias regime have mostly disappeared, whereas for the resonances in the positive-bias regime, the separation has increased. At the same time, the FG-dependent resonances remain mostly unaffected by the side-gate voltages. For *V*_SG1_ = *V*_SG2_ = –0.4 V, the low-bias gap is further increased and the FG-independent resonances mostly disappear for both negative- and positive-bias regimes. On the other hand, the FG-dependent ones, although still present, have reduced in intensity. Finally, for *V*_SG1_ = *V*_SG2_ = 1 V, the low-bias gap increases to 100 mV and the FG-independent resonances are absent, whereas the FG-dependent resonances have greatly reduced their intensity. Similar resonances are also observed in another device (D8) (Supplementary Section 3.6). To better visualize the effect of the side gates, Fig. 5d presents the d*I*/d*V* map as a function of *V*_Bias_ at a fixed *V*_FG_ = 2.7 V and also shown for four different SG settings (*V*_SG1_, *V*_SG2_) settings (Fig. 5c). Here the widening of the low-bias gap with increasing side-gate voltages is clearly visible, as well as the multiple resonances for *V*_SG1_ = *V*_SG2_ = –1.0 and –0.6 V. The spacing between the peaks varies between 24 and 39 mV.

Based on the multiple FG-dependent measurements we have performed, we attribute the FG-dependent resonances to states associated with the discrete energy levels of the GNR QD, as the FG is expected to couple more strongly to the GNR QD due to its close proximity. Conversely, the resonances that are largely unaffected by the FG originate from modulations in the DOS of the S-SWNT leads. This is in line with our previous observations in Fig. 5a,b. These lead states could originate either from the pristine S-SWNT or from localized states due to the presence of defects and other local barriers^48^. Based on the evolution of the FG-independent resonances with *V*_SG1_ and *V*_SG2_ (Fig. 5c), we are able to pinpoint that the transition from the bandgap to valence band of the S-SWNT electrode occurs between –0.6 and –0.4 V for both *V*_SG1_ and *V*_SG2_. The transition positions have been marked by white dashed lines in Fig. 5a,b. The position of the lead states is tunable by the SGs, which, in addition, also have the two following effects. First, the conductance through the S-SWNT is suppressed as the S-SWNT is being switched off. Second, when the resistance of the electrodes becomes comparable with that of the QD, the systems act as a voltage divider, with part of the bias voltage dropping across the electrodes themselves. As a result, the effective voltage across the QD is reduced and the low-bias gap increases (Fig. 5c). Finally, we notice that the FG-dependent resonances have predominantly positive slopes. We attribute this behaviour to asymmetric tunnelling rates between the GNR QD and leads.

Overall, by comparing M-SWNTs and S-SWNTs as electrode materials, we find that M-SWNTs have a clear advantage over the latter. First, no bandgap is present and the contacts can, therefore, not be switched off. In addition, devices with M-SWNT electrodes possess fewer signatures of the electrodes themselves in charge transport measurements. Moreover, M-SWNTs have been recently shown to be promising candidates for low-contact-resistance devices with two-dimensional semiconductors as the channel^49^. However, in our measurements, the formation of QDs points towards the presence of a large barrier at the SWNT–GNR interface. This manifests itself in currents that are up to tens of nanoamperes at 100 mV, about an order of magnitude lower than for GNRs contacted using palladium electrodes^28^. For improving the contacts, the use of intercalated metal adatoms between the two π-systems^50^ may be required. Finally, we note that even though the SWNT naturally allows for a single GNR to be contacted, as the films are grown in high density, we cannot exclude the scenario in which two GNRs in series are present in the nanogap, each connected to different electrodes. However, using our multigate architecture, this scenario can be identified, as it would show the characteristics of a double-QD system. Supplementary Section 5 presents such a case, with the observation of the characteristic high-bias triangles present in the maps of current as a function of *V*_FG_ and *V*_SG1_. Importantly, the bias triangles change direction on a reversal of the bias voltage—a key feature for double-QD systems.

## Conclusions

We have reported the contacting of individual on-surface synthesized GNRs using pairs of SWNTs (with diameters as small as 1 nm) as electrodes. The contacted GNRs exhibit behaviour that is characteristic of charge transport through a single QD, such as Coulomb blockade, excited states of vibrational origin and Franck–Condon blockade. DFT calculations highlight the importance of vibrational modes to electron transport in the SET regime of the GNR devices. Contacting single, long GNRs in a multigate architecture is important for exploiting their highly tunable physical properties in electronic and spintronic devices. In particular, GNR applications that require phenomena based on long-range effects—such as spin chains^29^ or the creation of topological bands due to the periodic placement of edge extension along the GNR backbone^4^—could benefit from the SWNT contacting method. These effects are promising for quantum technologies, such as quantum computing, quantum communication and energy conversion.

## Methods

### Fabrication and characterization

The device is fabricated as follows. First, a 100-nm-wide FG is fine patterned alongside the two side gates (SG1 and SG2). These gates consist of 5/15 nm of Cr/Pt and are covered by a 30 nm Al_2_O_3_ layer deposited using atomic layer deposition acting as the gate dielectric. A large-area quartz crystal (4 × 6 mm^2^) is used to synthesize a uniaxially aligned array of SWNTs, which is transferred on top of the aluminium oxide of the device chip using a wet-transfer method. The SWNT transistors are then fabricated by depositing a periodic array of metallic pads (3/50 nm of Cr/Pd) to contact the SWNTs. The overall channel length between the two metal electrodes is 2.5 μm. As the width of the metal contacts (10 μm) is comparable with the average separation distance between the SWNTs, we assume that most of the as-fabricated transistors contain only a single SWNT. Then, nanogaps of 15–25 nm are formed in the SWNTs using an optimized EBL process in combination with reactive ion etching. Here the electrode separation (15–25 nm) was set to be large enough to eliminate direct tunnelling contributions between the electrodes, but much smaller than the average length of 9-AGNRs. Finally, a dense array of uniaxially aligned 9-AGNRs is grown on a Au(788) substrate and transferred on top of the device^51^. The integrity of 9-AGNRs and their alignment with respect to the source–drain axis were confirmed using polarization-dependent Raman spectroscopy. Supplementary Sections 1.1 and 1.2 provide a more detailed description of the fabrication process.

Note that devices with both global back-gate architecture and multigate architecture were fabricated in this work. The electrical characterizations were performed at four different steps during the device fabrication using d.c. measurement techniques. First, after patterning electrode arrays in the transferred SWNT area, the gate-modulated electrical conductivity for each defined channel was measured. The purpose here was to screen the SWNT transistors and to determine the electrical properties of SWNTs at each transistor. Second, after forming the nanogaps on SWNT by EBL, the SWNT transistors were electrically characterized to ensure a clear separation between the electrodes. Devices with currents greater than 20 pA at *V*_Bias_ = 1 V were excluded from further characterization. Third, after the transfer of GNRs on SWNT electrodes, electrical measurements were performed to find devices bridged by GNRs, which were selected for low-temperature measurements. The electrical measurements for the first, second and third steps were performed at room temperature using an automatic probe station. Fourth, the preselected GNR devices were measured at a low temperature under vacuum conditions (<10^−6^ mbar). The global back-gate devices (D1 and D2) were measured in a commercially available cryogenic probe station (Lake Shore Cryogenics, model CRX-6.5K) at a base temperature of 9 K and the multigate devices (D3–D8) were measured in a commercially available ^3^He refrigerator (Oxford Instruments, model HelioxVL) at a base temperature of 255 mK. A data acquisition board (ADwin-Gold II, Jäger Computergesteuerte Messtechnik) is used to apply the bias and gate voltages and read the voltage output of the current–voltage converter (DDPCA-300, Femto Messtechnik).

All the devices were measured in a two-terminal setup, where we applied a bias voltage and measured the current, from which the differential conductance was calculated by taking the numerical derivative.

### SWNT growth, transfer and nanogap formation

The catalyst precursor was Fe(OH)_3_/ethanol solution with a concentration of 0.05 mol l^−1^ and the growth substrate was ST-cut quartz (single-side polished; miscut angle, <0.5°; surface roughness, <5 Å). After cleaning, the quartz substrates were annealed at 90 °C in air for 8 h for better crystallization. Before growth, the catalyst precursor was spin coated onto the substrates at a speed of 2,500 r.p.m. Then, the quartz substrates with a dispersed catalyst precursor were put into a one-inch tube furnace and heated in air to 830 °C. After the system was purged with 300 standard cubic centimetres per minute (s.c.c.m.) argon for about 10 min, a flow of hydrogen (200 s.c.c.m.) was introduced for 8 min to reduce the catalyst precursor to form Fe catalyst nanoparticles. Then, an extra argon flow (~50–150 s.c.c.m.) through an ethanol bubbler was introduced for the growth of SWNT arrays for about 15 min. After the growth, the tube was cooled to room temperature with argon gas protection. The density of the grown SWNT arrays could be adjusted by the flow rate of ethanol.

The transfer of SWNT arrays from the quartz substrate to the target substrate was conducted with the assistance of poly(methyl methacrylate) (PMMA). In detail, PMMA (*M*_w_ = 950,000) was spin coated onto the SWNT arrays at a speed of 3,000 r.p.m. and was heated to 150 °C for about 15 min. The PMMA film and encapsulated SWNT arrays were separated from the quartz substrate in a KOH aqueous solution (1 mol l^−1^, 70 °C). Then, the PMMA/SWNT film was attached to the target substrate and cleaned by ultrapure water. After drying at 60 °C for about 3 h, most of the PMMA was removed by hot acetone. The residual PMMA was further removed by decomposing at 450 °C in an argon and hydrogen atmosphere for 2 h.

To define the SWNT nanogaps, a 60-nm-thick CSAR resist (ARP 6200.04, Allresist) was spin coated. Following the second electron-beam exposure, the resist was developed using a suitable developer (AR 600-546, Allresist) at room temperature for 1 min followed by isopropyl alcohol rinse. Reactive ion etching (15 s.c.c.m. Ar, 30 s.c.c.m. O_2_, 25 W, 18 mtorr) for 12 s was used to cut the SWNT segment within the CSAR gap. After reactive ion etching, the etching mask was removed by immersing in 1-methyl-2-pyrrolidinone (Sigma-Aldrich) at room temperature for 10 min followed by 60 min at 80 °C, cooled down for 30 min, rinsed with isopropyl alcohol and blown dry with N_2_. This approach yields clean and well-separated SWNT electrodes (15–25 nm nanogaps). A similar process has been used to make clean graphene nanogaps, as reported elsewhere^19^.

### Characterization of SWNT nanogaps

The separation of SWNT electrodes (gap size) was assessed using scanning electron microscopy (Helios 450, FEI), and a high-resolution AFM instrument (Icon, Bruker) was employed to determine the electrode separation. The AFM instrument was equipped with a sharp cantilever (tip radius, 2 nm) (SSS-NCHR-20, Nanosensors) operated in the soft-tapping mode. The gap size is typically between 15 and 25 nm.

### On-surface synthesis of aligned 9-AGNRs and transfer to device substrate

The 9-AGNRs were synthesized from 3',6'-diiodo-1,1':2',1"-terphenyl (DITP)^52^. Using a Au(788) single crystal (MaTeK) as the growth substrate results in uniaxially aligned 9-AGNRs (GNRs grown along the narrow Au(111) terraces)^51^. The Au(788) surface was cleaned in an ultrahigh vacuum by two sputtering/annealing cycles: 1 kV Ar^+^ for 10 min followed by annealing at 420 °C for 10 min. Next, the precursor monomer DITP was sublimed onto the Au(788) surface from a quartz crucible heated to 70 °C, with the substrate held at room temperature. After the deposition of about 60–70% of one monolayer of DITP, the substrate was heated (0.5 K s^−1^) to 200 °C with a 10 min holding time to activate the polymerization reaction, followed by annealing at 400 °C (0.5 K s^−1^ with a 10 min holding time) to form the GNRs via cyclodehydrogenation. The average GNR length is between 40 and 45 nm (ref. ^52^). The 9-AGNRs were transferred from their growth substrate to the silicon-based substrates with predefined SWNT electrodes by an electrochemical delamination method using PMMA, as described previously^51,53,54^.

### Determination of electron–phonon coupling using Franck–Condon principle

From the Franck–Condon principle, the transition probability from the *N* state to the *N* + 1 charge state is given by the Franck–Condon factor^55^:

1 $${P}_{{{{{m,0}}}}}\propto {\left(\frac{{\rm{d}}I}{{\rm{d}}V}\right)}_{m}^{{\rm{max}}}\propto \frac{{\lambda }^{2m}}{m!}{{\rm{e}}}^{-{\lambda }^{2}},$$

where *m* is the difference in phonon quantum numbers.

### Computational methods

The optimized geometry, ground-state electron Hamiltonian and overlap matrix elements, as well as the phonon dynamical matrix of each structure studied in this paper, were self-consistently obtained using the SIESTA implementation^56^ of DFT. SIESTA employs norm-conserving pseudopotentials to account for the core electrons and linear combinations of atomic orbitals to construct the valence states. The generalized gradient approximation of the exchange–correlation functional is used with the Perdew–Burke–Ernzerhof parameterization and a double-zeta polarized basis set, a real-space grid defined with an equivalent energy cut-off of 250 Ry. The geometry optimization for each structure is performed to forces smaller than 20 meV Å^−1^. For the electron band structure calculation, the structure was sampled by a 1 × 1 × 20 Monkhorst–Pack *k*-point grid and assigned periodic boundary conditions in the *z* direction. For phonon band structure, we first displace each atom by 0.01 Å from their relaxed geometry in the positive and negative *x*, *y* and *z* directions and calculate the force matrix for each geometry. We used the VIBRA package of SIESTA to calculate the phonon band structure from the constructed force matrices. The discretization of all the energy bands^57^ for *ka* = [0, π] has been performed using Δ*ka* = 2π/*N* = 0.251. To calculate the electron transmission coefficient *T*(*E*), the mean-field Hamiltonian of 9-AGNRs with different lengths between SWNT electrodes were obtained from the converged DFT calculation and combined with GOLLUM^58,59^ implementation of the non-equilibrium Green’s function method. Supplementary Section 4 discusses the transmission through 9-AGNRs with SWNT electrodes.

## Supplementary information

Supplementary Information Supplementary Sections 1–5 and Figs. 1–12.

## Data Availability

The data that support the findings of this study are available via Zenodo at 10.5281/zenodo.7987323. Other data that support the findings of this study are available from the corresponding authors upon reasonable request.
