# Supplementary material for: Contacting individual graphene nanoribbons using carbon nanotube electrodes
Source: Nat Electron. 2023 Aug 14;6(8):572–81. doi: 10.1038/s41928-023-00991-3 (PMC10449622; doi:10.1038/s41928-023-00991-3)
Supplement: Supplementary file 1 — Supplementary Sections 1–5 and Figs. 1–12. [file 41928_2023_991_MOESM1_ESM.pdf]

nature electronics

Article

https://doi.org/10.1038/s41928-023-00991-3

Contacting individual graphene nanoribbons using carbon nanotube electrodes

In the format provided by the authors and unedited

CONTENTS

1. Materials,characterization and device fabrication 2
1.1. SWNT array, GNR array and their characterization 2
1.2. Process flow of multi-gate device fabrication 3
1.3. Room-temperature electrical characterizations of devices 5

2. Electron transport in global back-gate devices 6

3. Additional data for electron transport in multi-gate devices 7
3.1. D3: stability diagram recorded at $V_{SG1} = V_{SG2} = 0$ V 7
3.2. Low-temperature transport measurements for D4 and D5 with M-SWNT leads 8
3.3. Additional Franck-Condon analysis for D6 10
3.4. Additional QD analysis for D6 11
3.5. Tunnel coupling fitting 12
3.6. Low-temperature transport measurements for D8 with S-SWNT leads 14

4. Transmission through 9-AGNRs with SWNT electrodes 15

5. Double Quantum Dots in series 16

References 17

1. MATERIALS,CHARACTERIZATION AND DEVICE FABRICATION

1.1. SWNT array, GNR array and their characterization

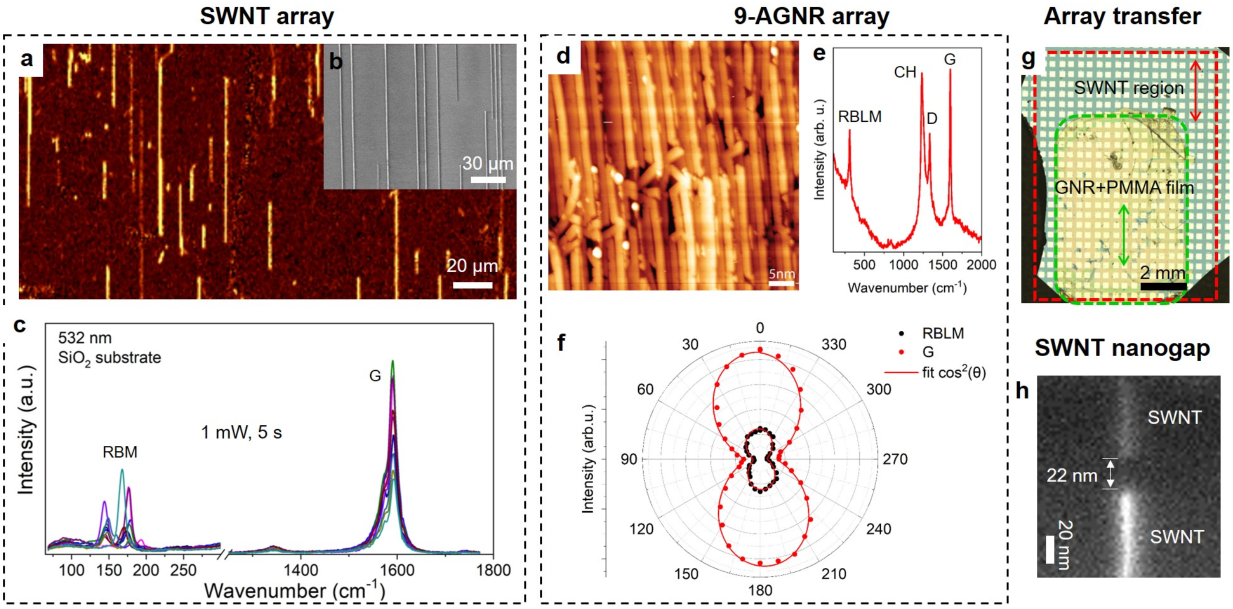

Figure 1. **Charaterization of aligned SWNT array, aligned 9-AGNR array, and their transfers.** (**a**) Raman map characterization of large-area uniaxially aligned SWNT arrays on SiO_2 substrate transferred from quartz crystal. (**b**) SEM characterization of the aligned SWNT arrays. The average separation distance of the SWNTs is $\sim$ 10 μm. (**c**) Raman characterization of the transferred individual SWNTs on SiO_2 substrate. The SWNTs used in this work have diameters of D = 1.5 ± 0.2 nm, as ditermined by the RBM peak statistics[1]. (**d**) Scanning tunneling micrograph (STM) of the 9-AGNRs on the Au (788) growth substrate, before transfer to the device substrate.The average GNR density is about 3 GNRs per terrace, with an average separation distance of 1.1 nm and an average length of 40-45 nm. (**e**) Typical Raman spectrum for 9-AGNRs on Pd substrate. The presence of the characteristic peaks (CH and RBLM) reveals the integrity of the 9-AGNRs upon annealing on the device substrate. (**f**) Polarization dependence of the G- and RBLM-peak intensity reveals almost perfect alignment with the source-drain axis. (**g**) Optical micrograph of a sample (global BG devices) with patterned SWNT electrode array and transferred GNRs/PMMA film. The GNR array is in parallel with the SWNT electrode array. (**h**) SEM characterization of a pair of representative SWNT electrodes with a gap size of $\sim$ 22 nm.

1.2. Process flow of multi-gate device fabrication

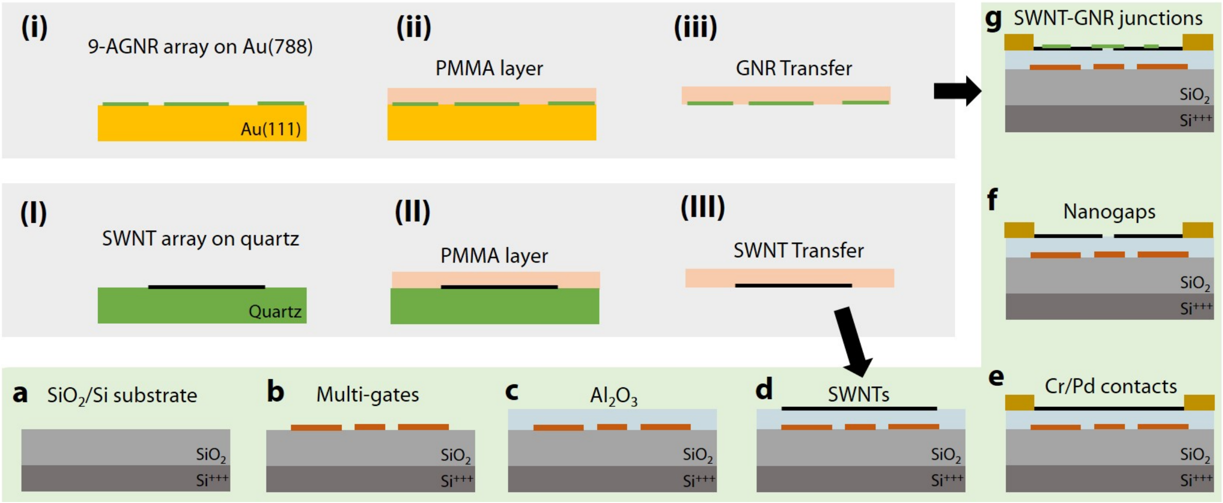

Figure 2. **Schematic illustration of the process flow to fabricate multi-gate SWNT-GNR junction devices.** (**i-iii**) On-surface synthesis of aligned 9-AGNRs and transfer to device substrate. (**I-III**) CVD synthesis of aligned SWNT arrays and transfer to device substrate. (**a-g**) Multi-gate substrate preparation and SWNT-GNR junction fabrication.

Figure 2 shows the detailed process flow for the multi-gate device fabrication. First of all, we use a cleaned silicon/silicon dioxide (285 nm) with predefined alignment markers as substrate (a). Gate electrode arrays with 3 electrodes (FG, SG1 and SG2 in Fig. S2a-b) per device (3 nm Cr / 15 nm Pt) are fabricated on the substrate using standard electron-beam lithography (EBL), electron-beam evaporation (EBE) and followed by a lift-off process, as shown in (b). The substrate with metal gate electrodes is then deposited an Al_2O_3 dielectric layer (30 nm) using atomic layer deposition (ALD) serving as a gate oxide (c). After that we spin-coated a 160 nm thick PMMA 950K (AR-P 672.02, Allresist GmbH) layer on the Al_2O_3 and used EBL to define an etch mask. A wet etching step of 30 minutes at room temperature using MF321 (DOW Chemical) opened the oxide on top of the gate for probing. The PMMA-based etch mask is removed using acetone and isopropanol followed by N_2 blow-dry. (I-III) show the growth of aligned SWNT arrays and transfer processes. Highly aligned SWNT arrays are grown on quartz wafers with relatively low coverage of Iron catalysts in a chemical vapor deposition (CVD) system. After growth, low-density ($\sim$ 1 SWNTs per 10 µm) horizontally aligned SWNT arrays are synthesized (I). Transfer of the SWNT arrays

is carried out by using a "wet" process. A 400 nm PMMA film is spincoated onto the as-grown SWNT arrays on quartz, and then the chip is immersed in an aqueous KON solution to etch the quartz. After releasing, the PMMA film with SWNT arrays is transferred on Al_2O_3 covered substrate (d). After the SWNT transfer, arrays of Cr/Pd (5 nm/60 nm) electrodes are fabricated for contacting SWNTs using standard EBL, EBE and followed by a lift-off process (e). The channel length is 2.5 µm while the channel width is designed as 10 µm for the purpose of contacting no more than 1 SWNT for each device. Afterward, a 60 nm thick CSAR resist (AR-P 6200.04, Allresist GmbH) is spin-coated, and followed by the electron beam exposure. Then the resist is developed using a suitable developer (AR 600-546, Allresist GmbH) at room temperature for 1 min followed by an IPA rinse. After reactive ion etching (15 sccm Ar, 30 sccm O_2, 25 W, 18 mTorr) for 12 s to define nanogaps (15-25 nm) at SWNTs, the etching mask is removed by immersing in 1-Methyl-2-pyrrolidinone (NMP) (Sigma Aldrich) for 60 min at 80 °C, and rinsed with IPA, and blown dry with N_2. This approach yields clean and well-separated SWNT electrodes (f). Finally, uniaxially aligned 9-AGNRs are synthesized on Au(788) single crystal (GNRs grown along the narrow Au(788) terraces) and transferred from their growth substrate to the silicon-based substrates with predefined SWNT electrodes by an electrochemical delamination method using PMMA as described previously[2–4].

1.3. Room-temperature electrical characterizations of devices

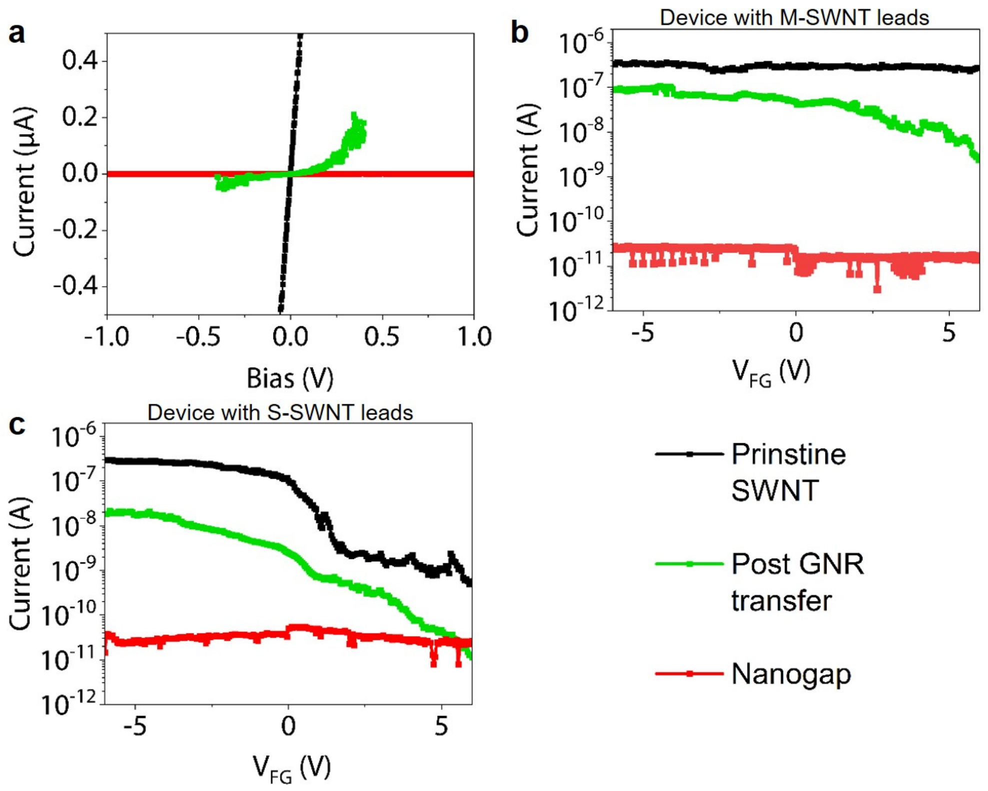

Figure 3. **Room-temperature electrical characterizations of devices for pristine SWNT, after gap formation, and after 9-AGNR transfer.** (**a**) IV for pristine SWNT (black curve), after gap formation (red curve), and after GNR transfer (green curve). The IV curves are acquired at zero gate voltage for all gates. (**b**) Current as a function of V_{FG} for pristine SWNT (black curve), after gap formation (red curve), and after GNR transfer (green curve) measured on a typical device with M-SWNT leads. After GNR transfer, the current show gate dependence because of the GNRs bridged in the channel and the surrounding GNR film. (**c**) Current as a function of V_{FG} for pristine SWNT (black curve), after gap formation (red curve), and after GNR transfer (green curve) measured on a typical device with S-SWNT leads.

2. ELECTRON TRANSPORT IN GLOBAL BACK-GATE DEVICES

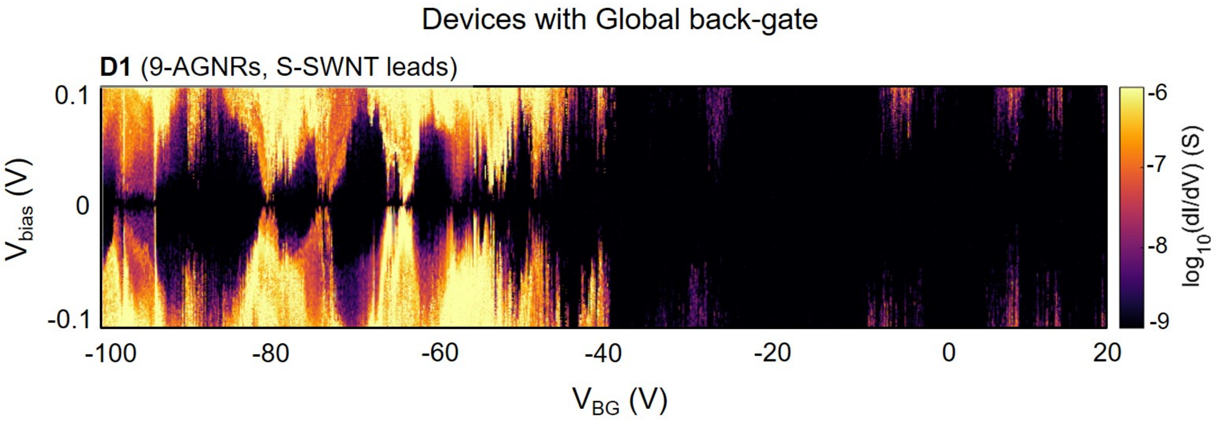

Figure 4. **Low-temperature transport measurements for D1 with global BG architecture.** Colour scale differential conductance versus V_{BG} and V_{Bias} for a 9-AGNR device (D1) with S-SWNT leads.

For the first stage, GNR devices with global back-gate (285 nm SiO_2) architectures were fabricated. In Fig. 4, the measurements for one 9-AGNR device (D1) with S-SWNTs as electrodes are shown. All measurements for global BG devices are performed in a cryogenic probe station with a base temperature of 9 K. Fig. 4 shows the plot of differential conductance versus V_{Bias} and global back-gate voltage (V_{BG}) of D1. In the V_{BG} range of -100 V $\sim$ -40 V, a few Coulomb diamonds with crossing points and sometimes "overlapping" features (e.g., at $\sim$ -90 V) are observed indicating the transport through a single 9-AGNR. When V_{BG} >-40 V, the conductance is pinched off due to the bandgap of S-SWNTs or 9-AGNRs. Here we realized that with a global BG architecture the chemical potential of GNRs and SWNT electrodes are tuned simultaneously by the common BG, then it is hard to distinguish the transport features between GNRs and SWNT electrodes via the bias spectroscopy.

3. ADDITIONAL DATA FOR ELECTRON TRANSPORT IN MULTI-GATE DEVICES

3.1. D3: stability diagram recorded at $V_{SG1} = V_{SG2} = 0$ V

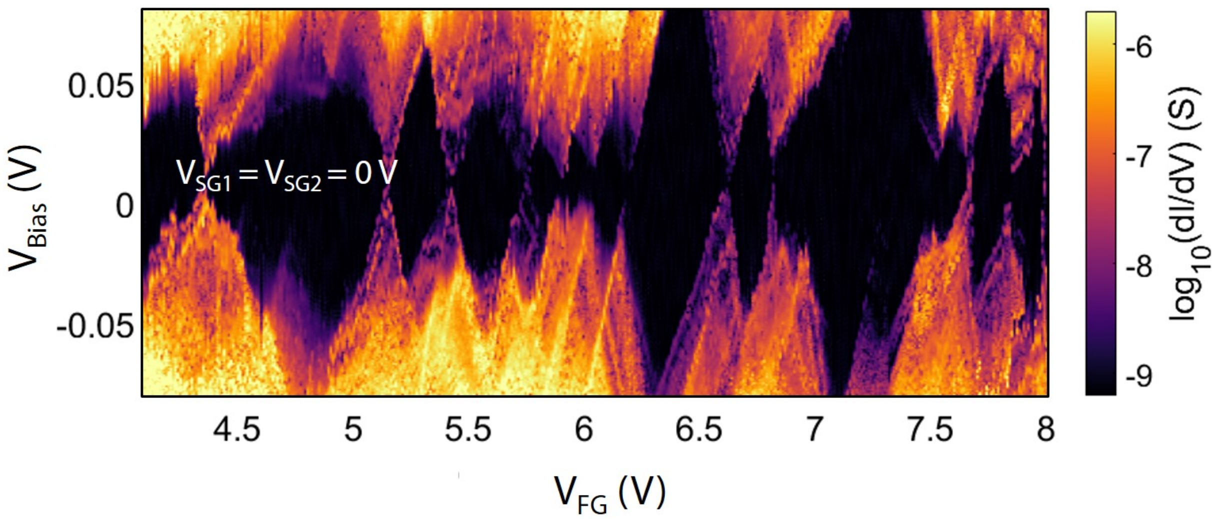

Figure 5. **Low-temperature transport measurements for multi-gate 9-AGNR devices (D3) with M-SWNT leads.** dI/dV as a function of V_{FG} and V_{Bias} (stability diagram) for fixed side gate voltages of $V_{SG1} = V_{SG2} = 0$ V.

3.2. Low-temperature transport measurements for D4 and D5 with M-SWNT leads

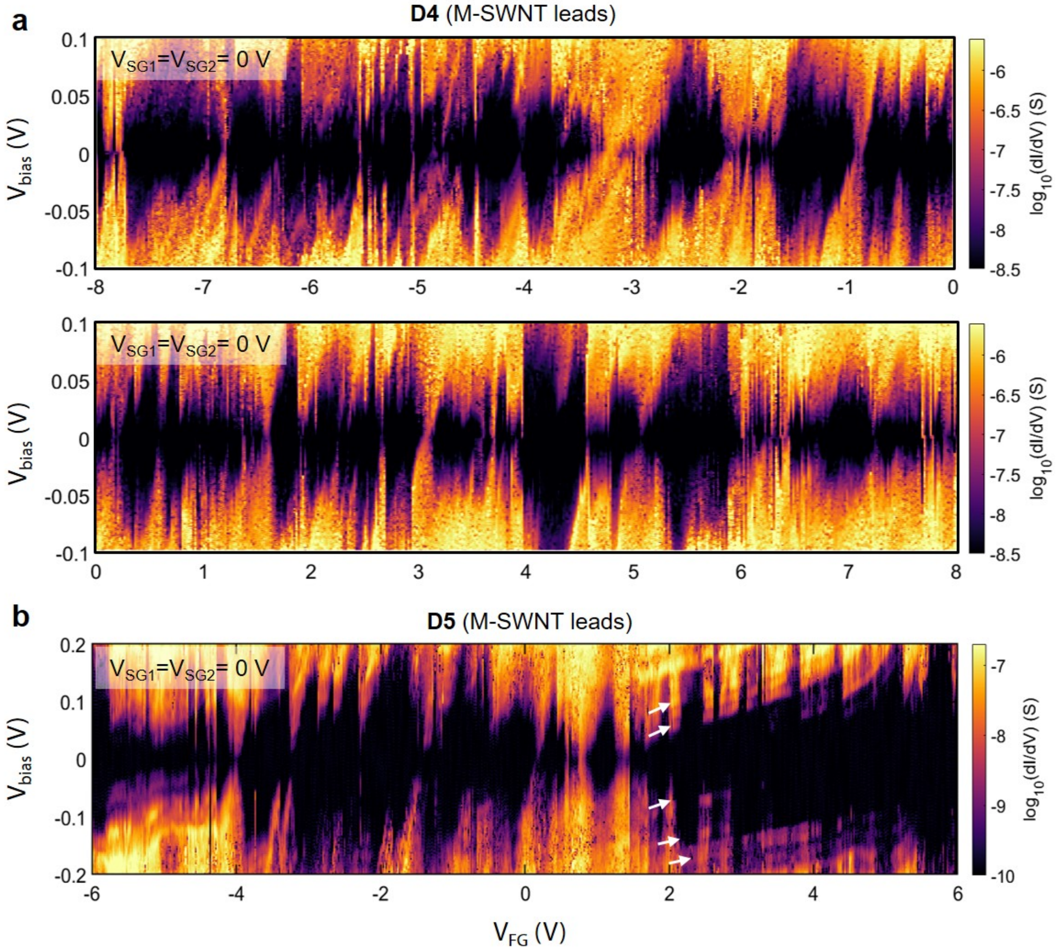

Figure 6. **Low-temperature transport measurements for multi-gate 9-AGNR devices (D4 and D5) with M-SWNT leads.** (**a**) Colour scale differential conductance versus V_{FG} and V_{Bias} for D4 with M-SWNT leads. The V_{SG1} and V_{SG2} are both fixed at 0 V. (**b**) Colour scale differential conductance versus V_{FG} and V_{Bias} for D5 with M-SWNT leads. The V_{SG1} and V_{SG2} are both fixed at 0 V. White arrows highlight the resonances originating from the modulation of DOS of M-SWNT leads.

In the given V_{FG} ranges, (-8 V, 8 V) for D4 and (-6 V, 6 V) for D5, multiple Coulomb diamonds are observed. However, these diamonds are irregular, aperiodic, and sometimes "overlapping" with addition energies varying between 50 meV and 100 meV for D4, and between 100 meV and 200 meV for D5. The diamond features most likely originate from

GNR QD with localized states formed at the GNRs due to local confinement potentials. For D5, in the V_{FG} range of 2 V to 6 V, several diagonal resonances at both the positive and negative V_{Bias} are observed. These resonances are attributed to the SWNT lead states that have a much smaller coupling to the FG than that of the GNR QD.

3.3. Additional Franck-Condon analysis for D6

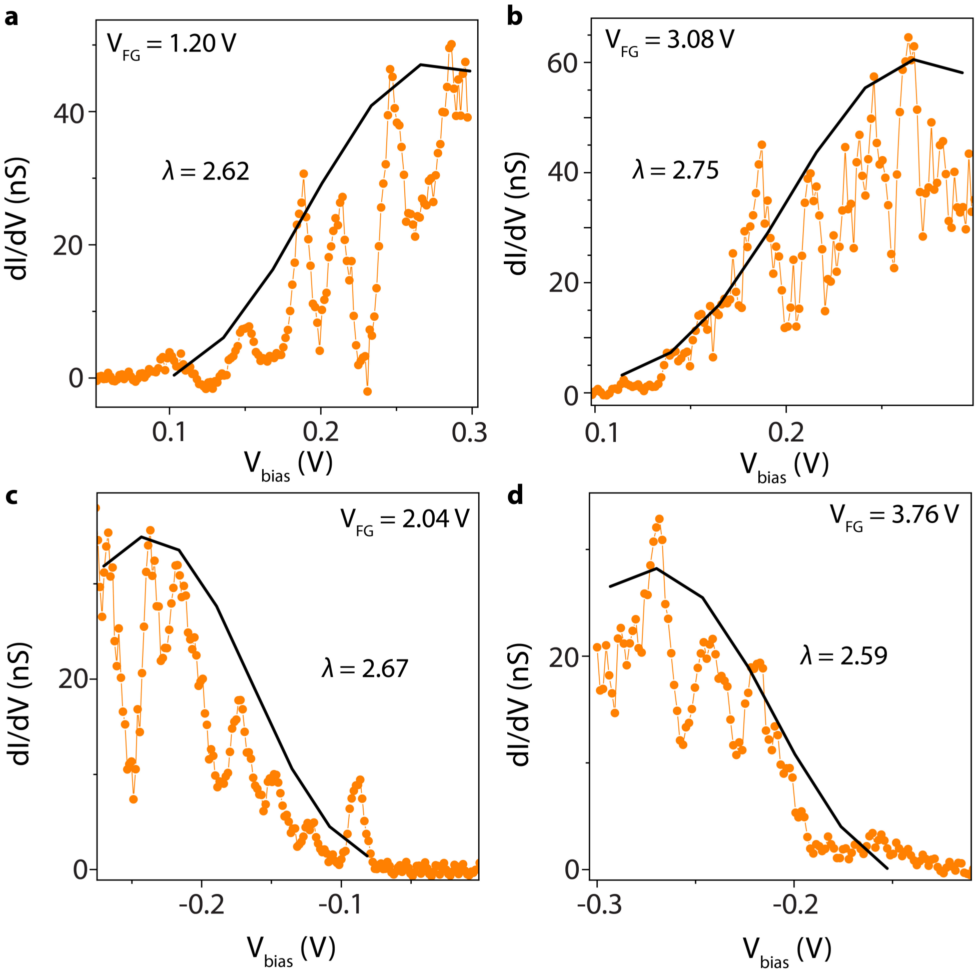

Figure 7. **Additional Franck-Condon analysis for D6.** Differential conductance traces measured at fixed V_{FG}: (**a**) 1.20 V, (**b**) 3.08 V, (**c**) 2.04 V and (**d**) 3.76 V. The black solid lines are fits to (dI/dV)max using the Franck-Condon progression described in the main text. The average value of λ is 2.66 ± 0.09.

3.4. Additional QD analysis for D6

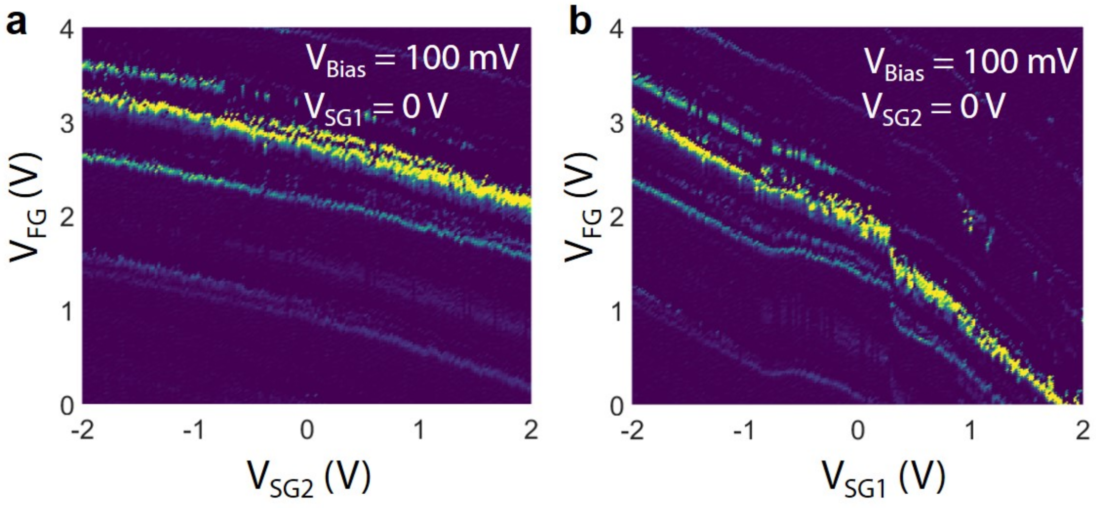

Figure 8. **Additional QD analysis for D6.** Map of the charge stability diagram in the (**a**) V_{FG}-V_{SG2} plane and (**b**) V_{FG}-V_{SG1} plane at V_{Bias}=100 mV, showing diagonal resonance features. To determine the position of the QD along the SWNT-GNR-SWNT channel, we have analyzed the respective gate coupling of the SG1, FG, and SG2 for device D6. Here shows conductance measurements as a function of the V_{FG} and V_{SG2} (left), and as a function of the V_{FG} and V_{SG1} (right) at V_{Bias} = 100 mV. From the change in the resonance position, we extract the following relative gate couplings $\alpha_{FG} : \alpha_{SG1} : \alpha_{SG2} = 1 : 0.81 : 0.29$. Based on these and position of the gates with respect to the SWNT-GNR-SWNT channel, we conclude that the QD is formed in the GNR, rather than the in SWNT leads.

3.5. Tunnel coupling fitting

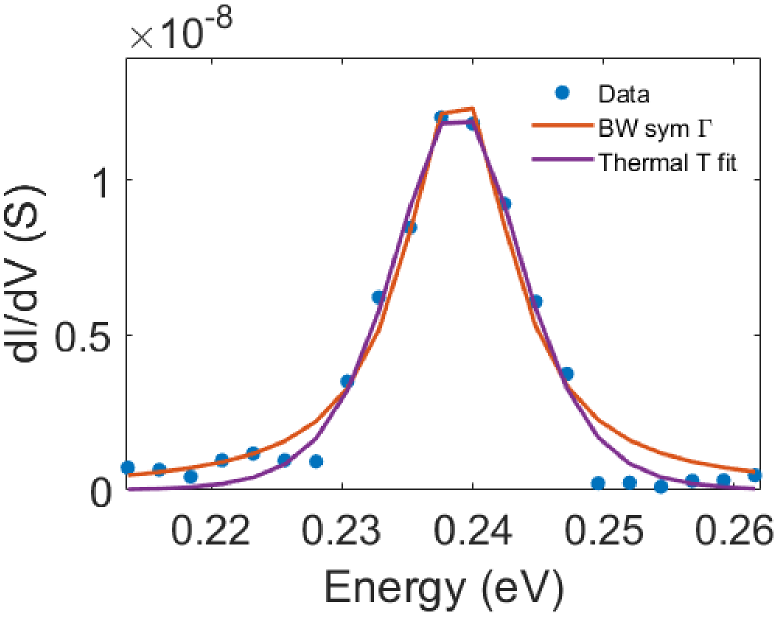

Figure 9. **Coupling fitting using Breit-Wigner model.** Fitting of a Coulomb peak at zero bias for one representative crossing point. The dI/dV data (blue points) are extracted from the differential conductance maps. The solid orange curve represents a fit to the Breit-Wigner (BW) model for resonant transport through a single-lifetime-broadened transport level.[5]. From the fitting, a coupling Γ of 4.9 meV is extracted. To exclude that the resonances are purely temperature-broadened, we fit our data to thermally broadened resonances (solid purple curve).[5] We find that the equivalent temperature is around $\sim$46 K, which is significantly higher than the cryostat temperature (255 mK). This indicates that the broadening of the resonances observed in our measurement is the result of the combined effect of hybridization with the electrode and temperature.

In the Breit-Wigner model, the peak shape is described by:[5]

$$G(\Delta V_G) = \frac{e^2}{h} \frac{\Gamma_1 \Gamma_2}{(\Delta E^2 + \frac{\Gamma^2}{4})}, \tag{1}$$

with $\Gamma = \Gamma_1 + \Gamma_2$ and the QD level detuning:

$$\Delta E = -e\alpha(\Delta V_G - V_G^{(0)}), \tag{2}$$

where $V_G^{(0)}$ is the position of the resonance. Here, α is the gate coupling of the backgate to the QD, described using the following relation:

$$\alpha = \frac{\Delta V_{Bias}}{\Delta V_G}. \tag{3}$$

The conductance of a thermally-broadened level is described as follows:[5]

$$G = \frac{e^2}{h}\frac{1}{4K_BT}\frac{\Gamma_1\Gamma_2}{\Gamma}cosh^{-2}(\frac{\Delta E}{2k_BT}), \tag{4}$$

with k_B being the Boltzmann constant and T, the bath temperature.

3.6. Low-temperature transport measurements for D8 with S-SWNT leads

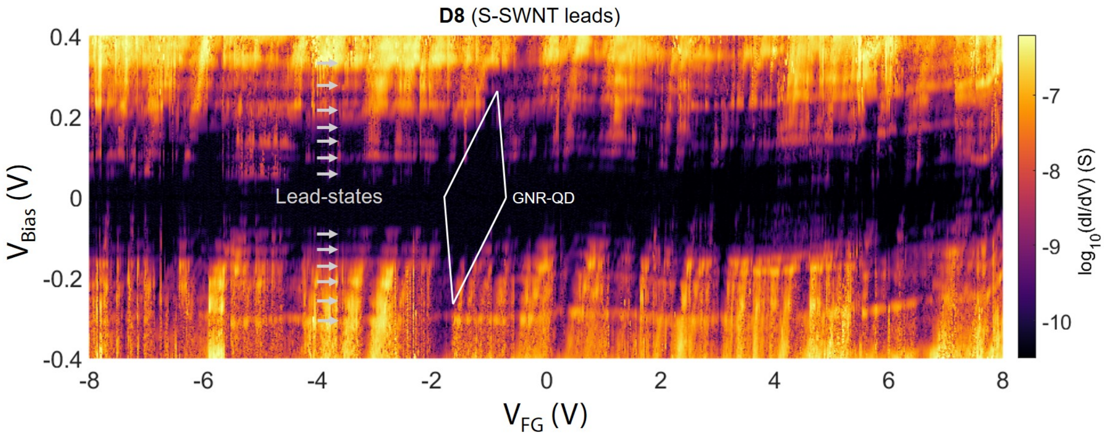

Figure 10. **Colour scale differential conductance versus V_{FG} and V_{Bias} at fixed V_{SG1}=V_{SG2}= 0 V for a 9-AGNR device (D8) with S-SWNT leads.** Several diamond-like features in the vertical direction are observed. Edges of one representative diamond are marked by white lines. We attribute these diamonds to the modulation of the discrete energy levels of GNR QD. In addition, several horizontal resonance lines in both positive and negative bias regimes are observed, as highlighted by grey arrows. We attribute these horizontal resonances to the modulation of the DOS of S-SWNT leads. One universal small bias gap is present in this device.

4. TRANSMISSION THROUGH 9-AGNRS WITH SWNT ELECTRODES

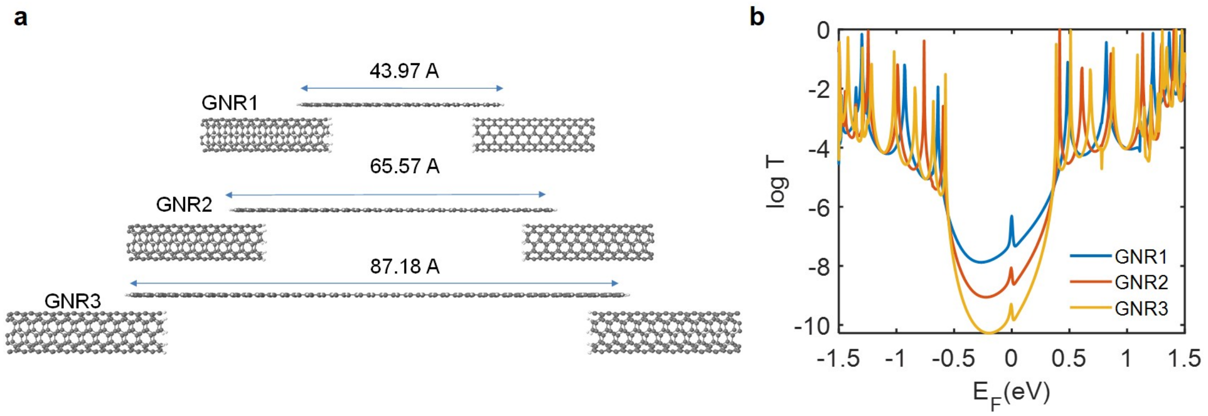

Figure 11. **Transmission through 9-AGNRs with SWNT electrodes.** (**a**) Structures of 9-AGNRs with SWNT electrodes. Three GNRs 1-3 with different lengths are illustrated. (**b**) Transmission curves of the junctions shown in **a** with varying GNR lengths.

5. DOUBLE QUANTUM DOTS IN SERIES

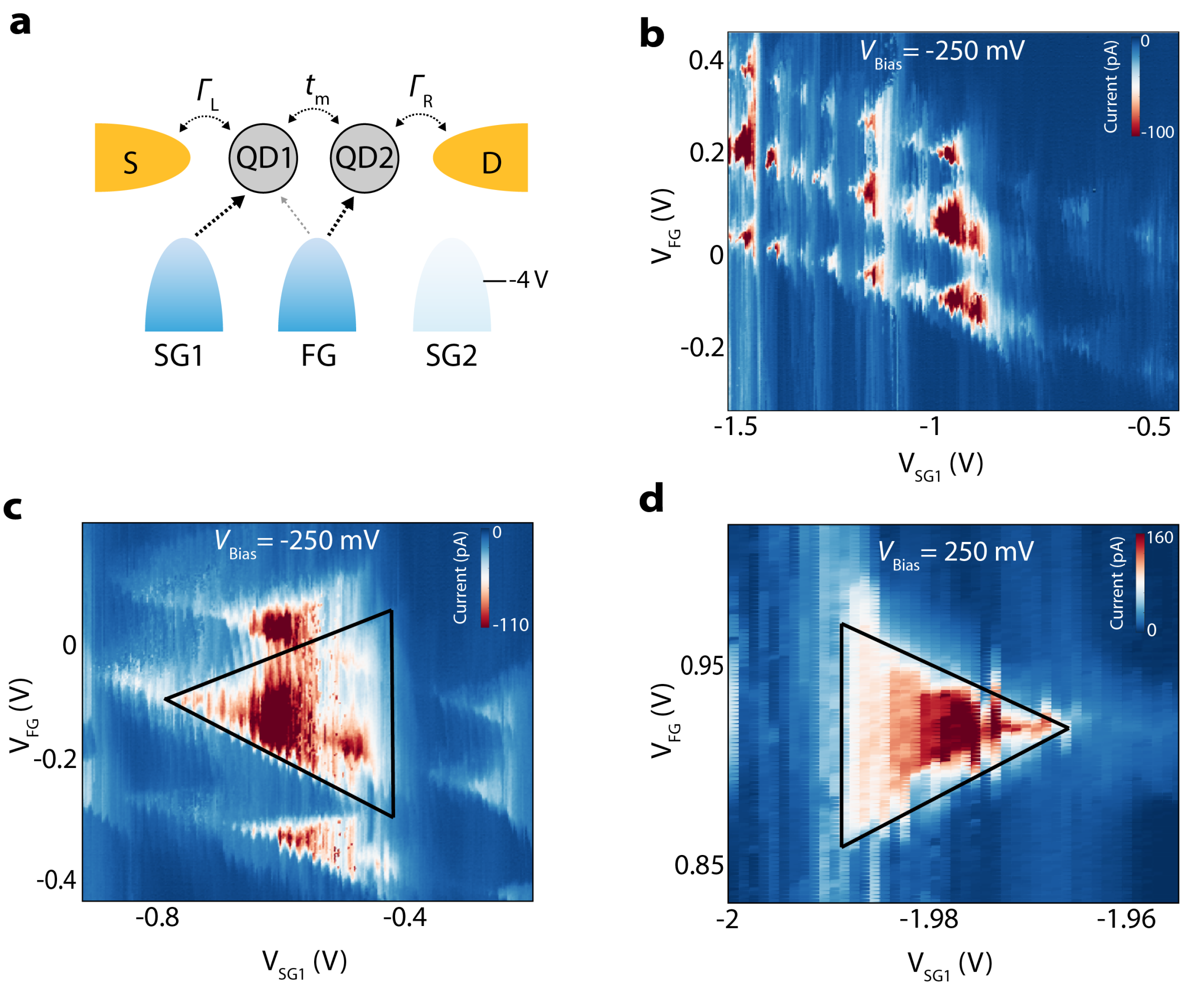

Figure 12. **Double QDs in series.** (**a**) Schematic model of the double QDs with two capacitively coupled gates (FG and SG1). (**b-d**) Current maps as a function of FG voltage and SG1 voltage (charge stability diagram) in the double QD regime at V_{SG2}=-4 V. (**b**) Overview map recorded at a negative bias of V_{Bias}=-250 mV. (**c**) High-resolution measurement recorded at negative bias V_{Bias}= -250 mV. (**d**) High-resolution measurement recorded at positive bias V_{Bias}= 250 mV.

A double quantum dot is formed in the nanojunction when two physical GNRs are connected in serial within the junction (as illustrated in Fig. 12a). Fig. 12b-d present several maps of the current as a function of the voltages on FG (V_{FG}) and SG1 (V_{SG1}). In Fig. 12b), an overview map is shown recorded at a negative bias voltage of V_{Bias}=-250 mV and V_{SG2}=-4V. The map shows a honeycomb-like pattern, characteristic for charge stability diagrams

of double QD systems. Here, the two gates FG and SG1 control the number of charges on dot 1 and 2, respectively. At low-bias, the corners of the honeycomb correspond to the triple points at which the energy level of the left dot aligns with the energy level of the right dots. As such, transport through the two QDs is allowed. As in our measurements the bias voltage is high, these triple points are expected to become triangular. Fig. 12c zooms in on a particular region of large current, highlighting the triangular shape of the triple points. This shape is characteristic for double QD systems. We note that as the bias voltage is large, some of the current triangles overlap with adjacent ones. Fig. 12d shows a representative triangle measured at a large positive bias of V_{Bias}=+250 mV. The bias triangles change direction upon a reversal of the bias voltage, a key feature for double QD systems. Altogether, our measurement are in line with the formation of a double QD system being formed on our junction, the presence of which is verified using a multi-gate measurement scheme.

---

[1] Q. Zhao and J. Zhang, Characterizing the chiral index of a single-walled carbon nanotube, Small **10**, 4586 (2014).

[2] J. Overbeck, G. Borin Barin, C. Daniels, M. L. Perrin, L. Liang, O. Braun, R. Darawish, B. Burkhardt, T. Dumslaff, X. Y. Wang, A. Narita, K. Müllen, V. Meunier, R. Fasel, M. Calame, and P. Ruffieux, Optimized substrates and measurement approaches for raman spectroscopy of graphene nanoribbons, Physica Status Solidi (b) Basic Research **256**, 1900343 (2019).

[3] O. Braun, J. Overbeck, M. El Abbassi, S. Käser, R. Furrer, A. Olziersky, A. Flasby, G. B. Barin, Q. Sun, R. Darawish, *et al.*, Optimized graphene electrodes for contacting graphene nanoribbons, Carbon **184**, 331 (2021).

[4] J. Overbeck, G. B. Barin, C. Daniels, M. L. Perrin, O. Braun, Q. Sun, R. Darawish, M. De Luca, X.-Y. Wang, T. Dumslaff, *et al.*, A universal length-dependent vibrational mode in graphene nanoribbons, ACS nano **13**, 13083 (2019).

[5] J. Gramich, A. Baumgartner, and C. Schönenberger, Resonant and inelastic andreev tunneling observed on a carbon nanotube quantum dot, Physical review letters **115**, 216801 (2015).
